# Recruitment of Patients With Amyotrophic Lateral Sclerosis for Clinical Trials and Epidemiological Studies: Descriptive Study of the National ALS Registry’s Research Notification Mechanism

**DOI:** 10.2196/28021

**Published:** 2021-12-07

**Authors:** Paul Mehta, Jaime Raymond, Moon Kwon Han, Theodore Larson, James D Berry, Sabrina Paganoni, Hiroshi Mitsumoto, Richard Stanley Bedlack, D Kevin Horton

**Affiliations:** 1 Agency for Toxic Substances and Disease Registry Centers for Disease Control and Prevention Atlanta, GA United States; 2 Sean M Healey & AMG Center for ALS Massachusetts General Hospital Boston, MA United States; 3 Spaulding Rehabilitation Hospital Harvard Medical School Boston, MA United States; 4 Department of Neurology Columbia University College of Physicians and Surgeons New York City, NY United States; 5 Department of Neurology Duke University School of Medicine Durham, NC United States

**Keywords:** amyotrophic lateral sclerosis, Lou Gehrig disease, motor neuron disease, clinical trials, patient recruitment, National ALS Registry, research notification mechanism

## Abstract

**Background:**

Researchers face challenges in patient recruitment, especially for rare, fatal diseases such as amyotrophic lateral sclerosis (ALS). These challenges include obtaining sufficient statistical power as well as meeting eligibility requirements such as age, sex, and study proximity. Similarly, persons with ALS (PALS) face difficulty finding and enrolling in research studies for which they are eligible.

**Objective:**

The aim of this study was to describe how the federal Agency for Toxic Substances and Disease Registry’s (ATSDR) National ALS Registry is linking PALS to scientists who are conducting research, clinical trials, and epidemiological studies.

**Methods:**

Through the Registry’s online research notification mechanism (RNM), PALS can elect to be notified about new research opportunities. This mechanism allows researchers to upload a standardized application outlining their study design and objectives, and proof of Institutional Review Board approval. If the application is approved, ATSDR queries the Registry for PALS meeting the study’s specific eligibility criteria, and then distributes the researcher’s study material and contact information to PALS via email. PALS then need to contact the researcher directly to take part in any research. Such an approach allows ATSDR to protect the confidentiality of Registry enrollees.

**Results:**

From 2013 to 2019, a total of 46 institutions around the United States and abroad have leveraged this tool and over 600,000 emails have been sent, resulting in over 2000 patients conservatively recruited for clinical trials and epidemiological studies. Patients between the ages of 60 and 69 had the highest level of participation, whereas those between the ages of 18 and 39 and aged over 80 had the lowest. More males participated (4170/7030, 59.32%) than females (2860/7030, 40.68%).

**Conclusions:**

The National ALS Registry’s RNM benefits PALS by connecting them to appropriate ALS research. Simultaneously, the system benefits researchers by expediting recruitment, increasing sample size, and efficiently identifying PALS meeting specific eligibility requirements. As more researchers learn about and use this mechanism, both PALS and researchers can hasten research and expand trial options for PALS.

## Introduction

Amyotrophic lateral sclerosis (ALS), commonly known as Lou Gehrig disease, is a progressive multifactorial neurodegenerative disease primarily affecting motor neurons. Conservative estimates suggest that approximately 17,000 Americans currently live with ALS, while 1500 new cases are diagnosed annually [1,2]. Most patients with ALS survive 2-5 years after receiving a diagnosis [3,4]. Although numerous treatments and therapeutic strategies are employed in the care of persons with ALS (PALS), there are only 2 approved medications that slow ALS progression: riluzole and edaravone. These drugs do not cure ALS, but rather modestly prolong survival or slow disease progression [5,6].

In 2008, the US Congress passed the ALS Registry Act which directed the federal Agency for Toxic Substances and Disease Registry (ATSDR) to create the National ALS Registry (Registry) [7]. The mission of the Registry is multifold and includes determining national epidemiological trends such as incidence, prevalence, and mortality; identifying and examining risk factors and potential etiologies; and facilitating and supporting ALS research [8].

Researchers face challenges in patient recruitment and enrollment, especially for rare, fatal diseases such as ALS [9]. These challenges include having sufficient sample size, satisfying narrow inclusion criteria (eg, disease duration <2 years, specific genetic mutations, forced vital capacity >70%), as well as meeting eligibility requirements such as age, sex, and study proximity [10-14]. Similarly PALS, like patients with other rare disorders, face difficulty finding and enrolling in research studies for which they are eligible [15]. In 2012, the Registry sought approval from the Centers for Disease Control and Prevention (CDC) Institutional Review Board (IRB) to use its database of patients as a recruitment tool for researchers [16]. The system is now called the research notification mechanism (RNM). The purpose of this system is to provide a tool for researchers to recruit for epidemiological, biomarker, and observational studies, as well as clinical trials. Our objective is to describe how ATSDR’s National ALS Registry is connecting PALS with opportunities to participate in ALS research and trials.

## Methods

Since its establishment in 2012, PALS enrolling in the ALS Registry have been provided the opportunity to consent to receive research notifications. Interested participants provide an email address for receipt of research notifications through the RNM. To use the RNM, ALS researchers seeking PALS for research or trial participation submit a completed application online at the National ALS Registry website [17]. In the application process, researchers can select specific eligibility criteria to screen patients. Such criteria could include age range, period since diagnosis, gender, family history of ALS, region (national, state, or city); however, some researchers may specify no screening criteria if their study could include all Registry participants (Table 1).

**Table 1:** Patient criteria under the National ALS Registry research notification application.

Prescreening	Criteria
Age range at diagnosis (years)	0-10, 11-20, 21-30, 31-40, 41-50, 51-60, 61-75, >75
Diagnosis years	From (yyyy) to (yyyy)
Gender	Male or female
City (State) of US residence	eg, Atlanta, Georgia
Family history of ALS^a^	Mother, father, brother, sister, children
No prescreening needed	Materials sent to all participants taking part in the process

^a^ALS: amyotrophic lateral sclerosis.

Proof of the researcher’s IRB approval is required; however, CDC IRB approval is not required as this system was previously approved. All applications are reviewed for completeness and researchers are contacted if items are outstanding (eg, missing curriculum vitae or application form). Applications are submitted to the Registry's review committee comprising of internal and external subject matter experts to determine scientific merit based on a set of established criteria (Table 2).

**Table 2:** Evaluation and approval of the application by the National ALS Registry review committee.

Evaluation	Criteria
1. Scientific merit (A-D is considered acceptable)	A. Outstanding/B. Excellent/C. Good/D. Acceptable/E. Unacceptable
2. Will the proposal provide useful information for ALS^a^ patients? (A-C is considered acceptable)	A. High potential/B. Strong potential/C. Good potential/D. Limited potential/E. No apparent potential
3. Are the patient contact procedures and materials clear?	Yes/No/Requires more information
4. Are the patient contact procedures and materials appropriate, necessary, and sufficient?	Yes/No/Requires more information
5. In your judgment, would most ALS patients find the demands of the protocol reasonable?	Yes/No/Requires more information
6. Is there an acceptable risk/benefit ratio?	Yes/No/Requires more information
7. Are there adequate protections for patient confidentiality and privacy?	Yes/No/Requires more information
8. Does the proposal reach a satisfactory threshold for all 7 criteria listed above?	Yes/No
9. Do you support approval of the proposal as it has been submitted?	Yes/No
Comments for the investigators (limit of 500 words)	

^a^ALS: amyotrophic lateral sclerosis.

**Figure 1:**
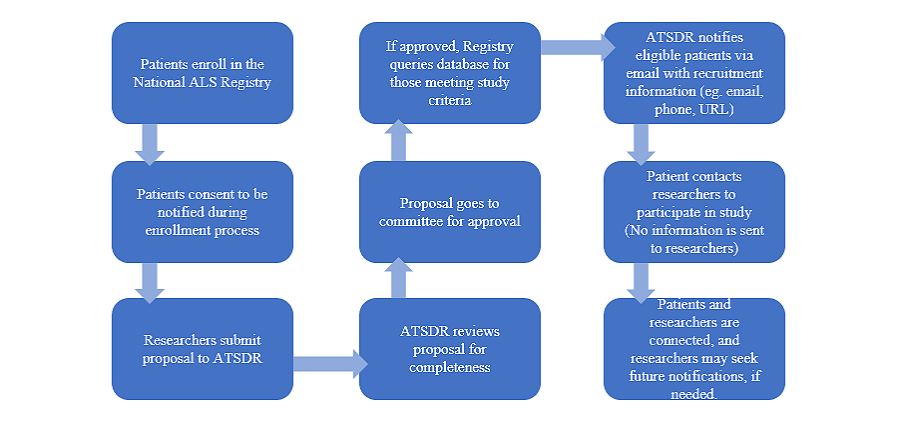
Flowchart of application approval process. ALS: amyotrophic lateral sclerosis; ATSDR: Agency for Toxic Substances and Disease Registry.

External committee members complete a conflict of interest form to ensure neutrality. Depending on the complexity of the application, approval time is within 3 weeks. If the committee cannot make a decision, final approval or disapproval resides with the ATSDR. Once the application is approved, Registry staff work with the researcher to coordinate a date and time for the notification, as shown in the process chart (Figure 1).

This communication helps the researcher to prepare for an influx of enrollment inquiries. The notification to PALS consists of a preapproved template with an attached document informing them of eligibility criteria (when applicable) and contact information for the researcher. A disclaimer is added to all notifications stipulating that CDC/ATSDR does not necessarily endorse the study and is not involved in its design or execution. PALS in receipt of the registration email can contact the research team directly to inquire about participation—no identifying information is shared with ALS researchers. RNM notifications can be sent to all registrants up to 3 times, if needed, and as requested by the researcher. In 2018, a fourth notification was implemented. The fourth communication follows the third round of notification to only newly registered patients after the 2018 implementation. All studies are posted on the Registry’s website and are classified as either “Active” or “Closed.” Contact information is also posted on the website for “Active” studies [18].

## Results

Since the launch of the Registry in 2010, over 90% of enrollees have elected to be notified about new ALS clinical trials and epidemiological studies. From 2013 to 2019, 46 institutions, both domestic and abroad, have leveraged this tool and 638,760 total emails were sent to consented patients. Annually, the greatest number of emails were sent in 2018 (n=293,422). In 2017, researchers at 10 institutions used the notification system—its broadest annual utilization. The median number of emails sent annually has been 41,433. The median number of emails per study was 8109 (Table 3).

**Table 3:** Number of emails sent to consented patients by Registry’s RNM^a^, 2013-2019.

Year	Studies (n=46), n (%)^b^	Emails (n=638,760), n (%)^c,d^	Yearly median
2013	4 (8.70)	5690 (0.89)	909
2014	8 (17.39)	27,035 (4.23)	5570
2015	9 (19.57)	41,433 (6.49)	5508
2016	3 (6.52)	20,007 (3.13)	2076
2017	10 (21.74)	82,114 (12.86)	9077
2018	6 (13.04)	293,422 (45.94)	30,091
2019	6 (13.04)	169,059 (26.47)	21,978

^a^RNM: research notification mechanism.

^b^Median number of studies for 2013-2019: 6.

^c^Median number of emails for 2013-2019: 41,433.

^d^Median number of emails per study: 8109.

Of the 7030 registrants who participated in the clinical notification, patients between the ages of 60 and 69 years had the highest level of participation (n=2417, 34.38%), whereas those between the ages of 18 and 39 and aged over 80 had the lowest (n=286, 4.07%, and n=218, 3.10%, respectively; Table 4). Age distribution in those who participated in clinical notification was found to be statistically different from the total registrants.

**Table 4:** Demographic characteristics of registrants who elected to receive notifications, January 1, 2013 to December 31, 2019.

Characteristic		Clinical notification participants (n=7030), n (%)	All registered participants (n=10,625), n (%)	*P* value^a^
**Age at diagnosis, n (%)**				<.001
	18-39	286 (4.07)	341 (3.21)	
	40-49	840 (11.95)	1041 (9.80)	
	50-59	2067 (29.40)	2782 (26.18)	
	60-69	2417 (34.38)	3817 (35.92)	
	70-79	1202 (17.10)	2198 (20.69)	
	80+	218 (3.10)	446 (4.20)	
**Gender, n (%)**				.71
	Male	4170 (59.32)	6289 (59.19)	
	Female	2860 (40.68)	4336 (40.81)	
**Census region, n (%)**				.18
	Midwest	2076 (29.53)	3315 (31.20)	
	South	2420 (34.42)	3480 (32.75)	
	West	1464 (20.83)	2216 (20.86)	
	East	1019 (14.50)	1556 (14.64)	
	Other/Missing	51 (0.73)	58 (0.55)	

^a^*P* value, Cochran–Mantel–Haenszel test.

More males participated (4170/7030, 59.32%) than females (2860/7030, 40.68%). Furthermore, participation of 7030 patients elected to be notified for clinical and epidemiological studies varied by region of country: South at 34.42% (n=2420), Midwest at 29.53% (n=2076), West at 20.83% (n=1464), and the East at 14.50% (n=1019; Table 4). This representation is in parallel with what has been observed in all registered participants. Based on anecdotal feedback from researchers, it is conservatively estimated that over 2000 patients were recruited for clinical trials between 2013 and 2019. This was calculated using an estimated average of 50 patients recruited per study for 46 studies or 2300 patients recruited since 2013. Some studies recruited more than 50 patients, whereas some less. Epidemiological studies conducted by Columbia University and University of Miami also demonstrated a higher percentage of patients recruited from the RNM than other sources (Tables 5 and 6).

**Table 5:** Utilization of the research notification mechanism by Columbia University^a^.

Enrollment method	Recruited (n=227), n (%)	Enrolled (n=103), n (%)
Email Blast (ATSDR^b^)	164 (72.2)	69 (66.9)
Pamphlet	21 (9.3)	13 (12.6)
Columbia University Irving Medical Center	35 (15.4)	20 (19.4)
ALSA^c^ listserv	1 (0.4)	0 (0)
ALS^d^ online forums	4 (1.8)	0 (0)
ATSDR conference	2 (0.9)	1 (1.0)

^a^Prospective comprehensive epidemiologic study in a large cohort in the National ALS Registry: identifying ALS risk factors.

^b^ATSDR: Agency for Toxic Substances and Disease Registry.

^c^ALSA: Amyotrophic Lateral Sclerosis Association.

^d^ALS: amyotrophic lateral sclerosis.

**Table 6:** Utilization of the research notification mechanism by the University of Miami^a^.

Enrollment method	Mean enrollment rate per month	Unpaired *t* test^b^ (*df*)	*P* value
Baseline	25		
Lecture	26	0.091 (17)	.92
Tweet	28	0.28 (22)	.78
Facebook	42	1.02 (20)	.32
National ALS^c^ Registry Research Notification Tool	89	2.94 (30)	.009

^a^Rare Disease Clinical Research Network, Contact Registry for the Clinical Research in ALS and Related Disorders for Therapeutic Development (CReATe) Consortium.

^b^Compared with baseline.

^c^ALS: amyotrophic lateral sclerosis.

A list of notable clinical trials and epidemiological studies is provided in Multimedia Appendix 1.

Since inception of the system, epidemiological studies have outnumbered clinical trials. These studies have focused on the evaluation of risk factors, genetics, and patient and caregiver burden, while clinical trials have focused on novel treatments to reverse or slow disease progression. A complete list of clinical trials and studies is available in the National ALS Registry website [18].

## Discussion

Clinical trial recruitment for rare diseases such as ALS has evolved. Researchers are continuously looking for novel methods for outreach and recruitment. The launch of the Registry’s RNM has helped to recruit patients for both clinical trials and epidemiological studies. The recruitment of participants for epidemiological studies and clinical trials is challenging [19,20]. This is especially the case for ALS where it is estimated that almost 60% of patients are not eligible for clinical trials [21]. The use of telemedicine to facilitate ALS clinical trials offers assistance with recruitment, consenting, and screening [22]. This is particularly evident with the COVID-19 pandemic and its impact on clinical research [23]. The cost of conducting clinical trials as well as the recruitment of patients are mitigating factors [24]. It is estimated that only 10% of the patient population with ALS participates in clinical trials [13].

Many factors contribute to the challenges of recruiting participants for ALS trials and a multifaceted approach is likely needed to improve research participation. Some models posit that behavior (trial enrollment) is determined by 3 main factors: motivation, access, and information [25]. Because ALS is a terminal disease, PALS are often highly motivated to enroll in trials. Unfortunately, access is still restricted for some ALS research. Almost 60% of PALS are deemed ineligible for most trials and access can be limited by mobility or geography [10,20]. The use of technology and the recent launch of the Healey ALS Platform Trial, which allows the testing of multiple treatments at once, should help reduce the cost of research, decrease trial time, and increase patient participation [26,27]. The Registry is poised to support this platform trial. This is evident by the growing list of clinical trials and epidemiological studies since 2013. The Registry’s novel system provides a user-friendly mechanism for researchers to access an existing pool of patients with ALS. Moreover, there is no charge to researchers to use the Registry to recruit patients with ALS.

Thousands of PALS have enrolled in research based on RNM notifications, and while the number of those who actually participate is challenging to determine with specificity, our estimate of 2300 PALS is based on conservative assumptions. In addition, the exact estimate of participants recruited by the Registry’s system is limited because many studies have more than 1 route of recruitment and researchers do not typically ask where a patient is recruited from such as ClinicalTrials.gov or the National ALS Registry. There were more males among the notification participants than females, in line with the demographics of ALS, which affects more males than females [2,8,28]. With over 8000 notification emails sent per study, the RNM allows researchers to access a large pool of potential participants quickly and efficiently. Furthermore, sending multiple notifications (n=4) allows patients who may have missed the notification message in their email inbox to be reminded of opportunity to participate in clinical trials and epidemiological studies. Because emails have inherent limitations and to increase durability of the recruitment message, the Registry has also posted all active studies with respective contact information on the website. This gives researchers the maximum exposure for their study.

The Registry has recruited for several notable clinical trials. These include 2 Phase 2/3 clinical trials, 1 utilizing mesenchymal stem cell–neurotrophic factor cells and the other a coformulation of sodium phenylbutyrate–taurursodiol, that have shown promise as recently reported [29,30]. In addition, the Registry has provided support for several epidemiological studies. Studies from Columbia University and the University of Miami demonstrated that the notification system led enrollment efforts when compared with other methods such as print and social media.

There are limitations to the RNM; for example, the system does not recruit for all ALS clinical trials because investigators must submit an application for consideration. Besides, the RNM only recruits patients who have self-enrolled in the Registry and not those who have been identified via Centers for Medicare & Medicaid Services (CMS) or Veterans Affairs (VA) databases. The Registry is working with partner organizations such as the ALS Association, Muscular Dystrophy Association, and the Les Turner ALS Foundation to increase enrollment, especially for minority patients.

Patient registries have been found to be successful in the recruitment of clinical trials for cancers and other conditions globally [31]. The National ALS Registry’s RNM is an effective tool for connecting PALS to researchers conducting clinical trials and epidemiological studies. The implementation and utilization of the RNM continues to benefit the researchers by helping to speed-up the recruitment process, increasing the study sample size, and easily and efficiently identifying patients meeting specific eligibility requirements. As more researchers learn about and use this mechanism, PALS and researchers can work together to accelerate therapy development for ALS.

## Appendix

Multimedia Appendix 1 Sampling of clinical trials and epidemiological studies utilizing the National ALS Registry's Research Notification Mechanism system, 2013-2019.

## Abbreviations

ALS: amyotrophic lateral sclerosis
ATSDR: Agency for Toxic Substances and Disease Registry
CDC: Centers for Disease Control and Prevention
CMS: Centers for Medicare & Medicaid Services
CReATe: Clinical Research in ALS and Related Disorders for Therapeutic Development
IRB: Institutional Review Board
PALS: persons with amyotrophic lateral sclerosis
RNM: research notification mechanism
VA: Veterans Affairs
